# The Combination of Computational and Biosensing Technologies for Selecting Aptamer against Prostate Specific Antigen

**DOI:** 10.1155/2017/5041683

**Published:** 2017-03-28

**Authors:** Pi-Chou Hsieh, Hui-Ting Lin, Wen-Yih Chen, Jeffrey J. P. Tsai, Wen-Pin Hu

**Affiliations:** ^1^Department of Bioinformatics and Medical Engineering, Asia University, Taichung City 41354, Taiwan; ^2^Department of Physical Therapy, I-Shou University, Kaohsiung City 82445, Taiwan; ^3^Department of Chemical and Materials Engineering, National Central University, Jhongli 32001, Taiwan; ^4^Department of Medical Laboratory Science and Biotechnology, China Medical University, Taichung City 40402, Taiwan

## Abstract

Herein, we report a method of combining bioinformatics and biosensing technologies to select aptamers against prostate specific antigen (PSA). The main objective of this study is to select DNA aptamers with higher binding affinity for PSA by using the proposed method. Based on the five known sequences of PSA-binding aptamers, we adopted the functions of reproduction and crossover in the genetic algorithm to produce next-generation sequences for the computational and experimental analysis. RNAfold web server was utilized to analyze the secondary structures, and the 3-dimensional molecular models of aptamer sequences were generated by using RNAComposer web server. ZRANK scoring function was used to rerank the docking predictions from ZDOCK. The biosensors, the quartz crystal microbalance (QCM) and a surface plasmon resonance (SPR) instrument, were used to verify the binding ability of selected aptamer for PSA. By carrying out the simulations and experiments after two generations, we obtain one aptamer that can have the highest binding affinity with PSA, which generates almost 2-fold and 3-fold greater measured signals than the responses produced by the best known DNA sequence in the QCM and SPR experiments, respectively.

## 1. Introduction

Prostate specific antigen (PSA) is a glycoprotein and has a molecular weight of 33-34 kDa. It is primarily created by prostate epithelial cells and is a serum marker commonly used to diagnose prostate cancer. Patients with prostate cancer generally have a large amount and a high concentration of PSA released into their circulatory system; as a result, their serum PSA concentration is 10^5^ times higher than that of regular healthy people. Thus, measuring serum PSA concentration is commonly used in the early diagnosis and for subsequent monitoring of patients with prostate cancer. Patients who undergo an examination and show a serum PSA concentration ≥ 4.0 ng/mL [1, 2] are suspected of having prostate cancer, after which doctors will ask the patients to have regular follow-up examinations. If the PSA concentrations of patients are over 10 ng/mL, the doctors will ask the patients to take a biopsy to confirm whether they truly have prostate cancer. PSA testing is crucial for determining whether an individual has prostate cancer, and most assays for PSA detection are based on the use of antibodies as the recognition elements [3].

In 1990, several research teams independently presented an in vitro selection technique. Such technique can find some certain nucleic acid sequences that can bind to target molecules of nonnucleic acids, in which the products demonstrate high affinity and specificity [3–5]. The technique is called the systematic evolution of ligands by exponential enrichment (SELEX). Concerning the SELEX technique, it is an iterative process and consists of three major steps: (1) binding; (2) partitioning; (3) amplification [6, 7]. The process starts from a pool of single-stranded oligonucleotide sequences incubated with the target molecule; some oligonucleotides exhibiting binding ability towards the target molecule can be obtained. In the partitioning step, the target-bound nucleic acids are separated from unbound sequences. Bound nucleic acids are then amplified to generate a new pool of nucleic acid sequences for use in the next round of selection. After repeating 8~20 cycles, nucleic acid aptamers with high affinity and specificity for the target molecule can be obtained. In essence, aptamers are short nucleic acid sequences with a length of 12 to 80 nucleic acids. Aptamers that have been identified by current research include RNA and DNA molecules, and RNA-type aptamers account for the majority of aptamers. Aptamers can be used to react with various target molecules such as metal ions, short peptides, pathogenic microorganisms, micromolecules such as amino acids, and macromolecules like proteins. Compared with antibodies, aptamers are manufactured by chemical synthesis; therefore batch-to-batch variation can be greatly minimized. The economical, high-accuracy large-scale production of aptamers makes aptamers very suitable for clinical applications [8]. In addition, nucleic acid aptamers feature advantages not found in common antibodies, which are listed as follows: small molecules, high stability, easy production, possibility for reuse, and unchanged affinity after simple chemical modifications [8]. Because of these advantages, aptamers can be used in various biotechnology-related fields such as environment and food quality tests, therapies, drug developments, purification processes, and diagnoses.

Previous studies have shown that the use of appropriate molecular simulation software is highly beneficial for designing molecules with high affinity and selectivity [9]. Advances in computer software and hardware over the past several decades as well as developments in mathematical models have resulted in increasing researchers using computers to simulate and predict the reactions of biomolecules and chemical molecules. Anderson and Mecozzi [10] employed the molecular simulation method in addition to conducting traditional experiments to investigate the interaction of the 35-mer aptamer and cofactor flavin mononucleotide (FMN). Their study successfully reduced the length of the 35-mer aptamer to 14 mers while retaining the binding ability for flavin mononucleotide.

In 2011, Bini et al. [11] proposed a computationally assisted method to study and evaluate aptamer-protein interaction for selecting new aptamer sequences. They performed molecular simulations by using a molecular model and conducted an experiment to verify the prediction results of the molecular simulations. Bini et al. initially used a well-known 15-mer thrombin binding aptamer (TBA) and then they modified base sequences at certain locations to generate a variety of mutant aptamer sequences. After docking simulations, experiments were performed by using a surface plasmon resonance (SPR) biosensor. The results indicated a mutant sequence could have a slightly better performance than the well-known TBA, and they found that experimental results were in agreement with the simulation findings. Lupold et al. [12] reported the specific binding between an RNA aptamer and a prostate specific membrane antigen (PSMA); the RNA aptamer demonstrated the potential to be used as a drug carrier to treat prostate cancer. In 2010, Jeong et al. [13] researched and found an RNA aptamer that can be used to identify PSA; the RNA aptamer featured a 5′-end to 3′-end sequence of CCGUAGGUCACGGCAGCGAAGCUCUAGGCGCGGCCAGUUGC. Savory et al. [14] selected five DNA sequences by using the SELEX technique and performed subsequent in silico analyses. With the use of selected five DNA sequences as parent sequences, the genetic algorithm (GA) was adopted to generate next-generation sequences and those generated sequences were synthesized and assayed in vitro. One of the fourth-generation DNA aptamers exhibited a 48-fold higher PSA-binding ability than the parent sequences.

Some scholars have identified RNA aptamers as a possible alternative to antibodies for testing serum PSA concentration [12, 13]. Nevertheless, the long length of RNA aptamers could cause the difficulty in the commercial synthesis and limit the applications [3]. By contrast, DNA-based PSA aptamers had been used to detect PSA by using different sensing technologies [15, 16]. Herein, we reported a study that employed computational approaches, including GA, the analysis of DNA secondary structure, and the molecular simulation, to evaluate the aptamer-protein interactions and biosensing technologies to verify the PSA-binding ability of selected aptamers. The original five sequences of aptamers reported in the study of Savory et al. [14] were chosen as the parent sequences and the genetic algorithm was used to generate new nucleic acid aptamers for the selection. By using the proposed strategy of selection, a new DNA aptamer exhibited stronger binding capability with PSA which was selected in the end of selection procedure. We think the proposed strategy of combining computational approaches (GA and molecular simulations) in the selection of aptamer can streamline the number of experiments carried out for the selection, and the results show that it is possible to complement SELEX for the selection of aptamer.

## 2. Materials and Methods

### 2.1. The Overview of Research Process

**Figure 1:**
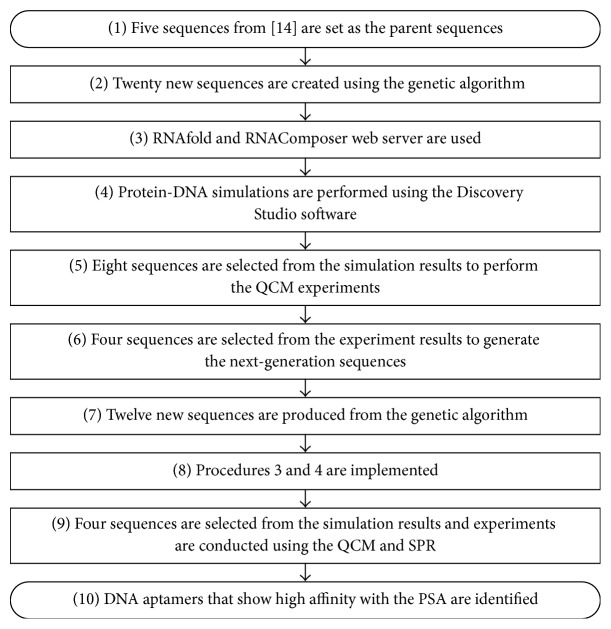
Flow chart of the study.

Figure 1 shows all steps of aptamer selection in this study. First, five sequences that can bind to PSA (obtained by Savory et al. by using the SELEX procedure [14]) were set as the parent sequences; Savory et al. named the five sequences as PSap#4-3, PSap#4-4, PSap#4-11, PSap#4-9, and PSap#4-6. To enable the genetic algorithm to calculate the four bases used in this study (i.e., A, T, G, and C), such bases were coded; bases A, T, G, and C for the five sequences were numbered 00, 01, 10, and 11, respectively. The genetic algorithm toolbox for MATLAB® [17] was utilized to perform reproduction and crossover operations for generating 20 next-generation sequences. The sequence information (in numerical format) was then converted back to the original base sequence format by using a C# program written by us. The detailed information of generating new sequences was provided in the Section S1.1 of Supplementary Material, available online at https://doi.org/10.1155/2017/5041683. The computational processes of using genetic algorithm were the same in the generation of two next-generation sequences. Next, an RNAfold web server [18] was used to predict and analyze the DNA secondary structure of the 20 sequences, in which sequences that failed to form a clear secondary structure were removed. The RNAfold web server is suitable for predicting secondary structures of DNA and RNA sequences. Then, an RNAComposer web server [19] was utilized to build three-dimensional molecular models for the remaining DNA aptamers. The method used by the RNAComposer web server is based on the machine translation principle and needs the information of secondary structure provided by RNAfold, and it operates on the RNA FRABASE database. After getting the RNA model generated by the RNAComposer web server, we used Accelrys Discovery Studio (DS) 4.1 to edit the pyrimidine bases in the structure file to make uracil become thymine. These DNA models were used for subsequent docking calculations in molecular simulations. We obtained the three-dimensional structure of PSA from a molecular structure file numbered “3QUM” in the Protein Data Bank (PDB). The 3QUM contained not only the molecular structure of PSA, but also that of Fab fragments of two monoclonal antibodies (antibodies were numbered as “5D5A5” and “5D3D11”). DS 4.1 was utilized to carry out molecular simulations. The DS software was used to read the molecular structure files (3QUM), and structures of two antibodies were removed and the structure of PSA was retained for subsequent simulations. This study used a docking program ZDOCK and the ZRANK scoring function to assess the interactions between the DNA aptamers and PSA. According to the simulation results of first-generation sequences, eight DNA aptamers were selected and synthesized for the quartz crystal microbalance (QCM) experiments. QCM experiments were performed to evaluate the real binding situations between the eight selected sequences of the first round and PSA. On the basis of the experiment results, four sequences were chosen out of the eight as the parent sequences to produce second-generation sequences. A genetic algorithm was utilized again to perform reproduction and crossover operations, which generated 12 second-generation sequences. The RNAfold web server was used to analyze the secondary structure for each second-generation sequence, and then an RNAComposer web server was employed to build 3D structural models of selected aptamers for molecular simulations. Finally, three aptamer sequences selected from the simulations were used to perform the QCM and SPR experiments for the assessment of aptamer-PSA interactions. Besides, an aptamer named as ΔPSap4#5 in the Savory et al.'s study [14] showed the highest PSA-binding ability was used as the control groups in the QCM and SPR experiments. All QCM and SPR experiments for each aptamer were done in triplicate.

### 2.2. Molecular Simulations

The ZDOCK simulation function offered by the DS software was used to assess the interactions between the DNA aptamers and PSA. ZDOCK is a docking method commonly used in protein-protein interaction simulations. The ZDOCK scoring function evaluates the protein-protein interactions by taking shape complementary (SC), electrostatics, and pairwise atomic potential into consideration. According to our previous studies [20, 21], ZRANK score was more suitable for using in the evaluations of the interactions between proteins and aptamers with longer sequences in length, which was due to the results of ZRANK scores that were closer to those obtained from experiments. This ZRANK program with an optimized energy function can significantly improve the success rate of prediction from the initial ZDOCK [22]. Regarding the equipment used to perform the calculations in this study, it comprised HP Z620 desktop workstation, two Intel® Xeon processors (containing 24 computing cores), a memory of 36 GB, and a 64-bit Windows 7 Professional Operating System.

### 2.3. Reagents and Biological Molecules

We purchased the poly(ethylene glycol) thiol (Thiol-PEG4-Alcohol) from Broadpharm Inc. (San Diego, California, USA). All of the DNA aptamers were synthesized and purchased from MDBio, Inc. (Taipei City, Taiwan), and each sequence was modified with 1-hexanethiol (C_6_SH) at the 5′ end. The human prostate specific antigen was obtained from MP Biomedicals, LLC (Santa Ana, California, USA). Sodium dihydrogen phosphate monohydrate (KH_2_PO_4_) and sodium chloride were purchased from Sigma-Aldrich Co., LLC (St. Louis, Missouri, USA). Dibasic sodium phosphate (Na_2_HPO_4_) and potassium chloride were acquired from J.T. Baker (Center Valley, Pennsylvania, USA). Phosphate-buffered saline (PBS) solution with 1x concentration was used in the experiments, which contains 10 mM dibasic sodium phosphate, 137 mM sodium chloride, 2 mM potassium dihydrogen phosphate, and 2.7 mM potassium chloride (adjusted to pH 7.4 with HCl). All other chemicals used in this study were of reagent grade.

### 2.4. Quartz Crystal Microbalance (QCM) Instrument

After structural analysis and simulations were completed, QCM experiments were used to evaluate the binding reactions between the DNA aptamers and PSA. The amount of change in frequency signals caused by the binding reactions was used to identify which DNA aptamers displayed favorable binding ability with PSA. The QCM instrument is produced by CH Instruments, Inc. (USA) and the model is CHI 410C. The variations in the crystal oscillator frequency of the QCM were primarily related to changes in the mass on the sensor surface. The relationship formula between oscillation frequency and adsorption quality is expressed in

(1) $$\begin{array}{c} \Delta f=-\frac{2{f_{0}^{}}^{2}\Delta m}{\left[Asqrt\left(\mu \rho \right)\right]}, \end{array}$$

where *f*_0_ is the fundamental resonant frequency of crystal, *A* is the area of the gold disk on the crystal (0.196 cm^2^), *ρ* is the density of crystal (2.684 g/cm^3^), and *μ* is the shear modulus of quartz (2.947 × 10^11^ g/cm*∗*s^2^).

### 2.5. Surface Plasmon Resonance (SPR) Instrument

The SPR imaging (SPRi) platform used in this study is developed by the Institute of Photonics and Electronics (IPE, Prague, Czech Republic) [23, 24]. This SPR platform is very sensitive and is capable of detecting the change of refractive index unit (RIU) occurring on the sensing surface better than 10^−6^. The p-polarized light beam with a central emission wavelength of 750 nm illuminates on the chip and excites surface plasmon waves at the metal-dielectric interface. For this SPR instrument, the amount of biomolecular interactions on the sensing surface can be expressed as biomolecular surface coverage. The smallest amount of biomolecular surface coverage that can be detected is 0.02 ng/cm^2^. The SPRi can perform traditional measurements or carry out a high-throughput measurement by using an array chip. The SPR chips were fabricated by precoating a thin layer of chromium (thickness approx. 2 nm) on the BK7 glass substrate, and a layer of gold film (thickness approx. 48 nm) was coated via an evaporation deposition process afterward. All SPR experiments were performed at a controlled temperature of 25°C with a constant flow rate of 50 *μ*l/min. The total amount of flow channels in the SPRi is 6, and one of the channels is utilized as the reference channel. After subtracting the data of reference channel, experimental data of aptamer-protein interactions could be obtained from other five channels.

### 2.6. Surface Functionalization and Binding Experiments

Prior to performing the experiment, the quartz crystal chip underwent a modification treatment. PEG thiols with an OH functional group and synthesized DNAs were all dissolved in a 1 M KH_2_PO_4_ solution to the concentrations of 19 mM and 0.2 *μ*M, respectively. Appropriate amounts of OH-PEG thiol and the synthesized DNA were taken and mixed together to make the DNA have a final concentration of 100 nM. The PEG thiol in the mixed solution had a concentration of 5 *μ*M and the molar ratio of DNA/PEG was 1 : 50. Next, 0.3 ml of DNA/PEG mixed solution was applied to cover the gold film on the surface of the quartz crystal chip. The chip was placed in a Petri dish and sealed with parafilm to react for 24 h in a 4°C environment. After the reaction, a DNA/PEG self-assembled monolayer (SAM) formed on the chip surface. The PEG resisted and lowered the nonspecific adsorption of proteins as well as providing enough space for preventing three-dimensional steric hindrances during molecular interactions. The modified chip was initially installed inside a chamber in the QCM measurement. Initially, 3.3 ml of 10 mM PBS (pH 7.4) solution was added to the QCM chamber. After the oscillation frequency of the chip stabilized, 0.5 ml of PSA solution (2 ng/ml) was injected into the device to observe the binding reaction of the PSA and the aptamer immobilized on the chip surface.

Concerning the experiment performed on the surface plasmon resonance biosensor, the gold film on the chip was treated in a manner similar to that on the QCM chip. The surface functionalization of gold film on the chip was accomplished by directly immersing in the DNA/PEG mixed solution for 24 h at 4°C. The chips were then rinsed with DI water and blown dry with nitrogen. Afterwards, the SPR chip was installed in the SPR instrument and 10 mM PBS was introduced to flow channels through a peristaltic pump. When the instrument displayed the SPR signal reaching a stable state, the solution containing 10 mM PBS and PSA with the concentration of 2 ng/ml was injected for 30 min. Subsequently, PBS solution was again introduced to flow channels for 13 min to obtain the final reaction amount from the specific binding of the aptamer and PSA. The affinity and kinetic parameters of aptamer-protein interactions were obtained by using two mathematical equations based on the first-order kinetics model for fitting SPR sensorgrams [20, 25]. From the calculation, the association rate constant *k*_*a*_ and the dissociation rate constant *k*_*d*_ can be determined. In addition, the binding affinity, *K*_*A*_, is defined in accordance with the relationship *K*_*A*_ = *k*_*a*_/*k*_*d*_.

## 3. Results and Discussion

### 3.1. First-Generation Sequence Calculations and Molecular Simulation Results

Calculations were made using a genetic algorithm, which produced 20 sequences as shown in Table 1. The third and fourth column of Table 1 are minimum free energy (kcal/mol) and dot-bracket format, respectively, which were obtained by performing analyses from the RNAfold web server. Sequences with a dot-bracket format comprising exclusively periods (e.g., PSAG16) signified that such sequences did not display a clear secondary structure. In other words, they did not have a clear stem-loop structure and were not appropriate aptamer candidates. For such sequences, no next-stage molecular simulation analysis was performed. For other sequences, 3D structural models were built using the RNAComposer web server and these models were then edited in the DS software. Graphic results of 3D structural models built by the RNAComposer web server and edited by the DS software are shown in Figure S1 (in the Supplementary Material). The structural models of aptamers and the PSA then underwent ZDOCK simulations, with each molecular simulation calculation lasting approximately 15 h long. The simulations produced ZDOCK and ZRANK scores for these sequences, in which those with a lower ZRANK score indicated a superior docking result. According to the molecular simulations, first-generation aptamers that showed the most favorable binding capability with PSA, listed in descending order, were PSAG11, PSAG114, PSAG119, PSAG118, PSAG112, PSAG117, PSAG15, and PSAG17.

### 3.2. QCM Experimental Results of the First-Generation Sequences

The eight sequences described in Section 3.1 (i.e., PSAG11, PSAG15, PSAG17, PSAG112, PSAG114, PSAG117, PSAG118, and PSAG119) were sent to a vendor to synthesize before QCM experiments were conducted. The experimental results are shown in Table 2 and indicate that the eight sequences can bind to PSA, resulting in relatively larger changes to the QCM oscillation frequency (i.e., drops in oscillation frequency). The changes in QCM oscillation frequency generated by the sequences, listed in descending order, were PSAG15, PSAG119, PSAG117, PSAG112, PSAG114, PSAG17, PSAG118, and PSAG11. However, most of ranking results from experimental and simulation studies are not consistent. Concerning the ranking of the eight sequences in experiments, only PSAG112 and PSAG119 showed a ranking that closely matched those obtained from the molecular simulations. One of the reasons is that we supposed that the docking method used in this study is not the optimal algorithm for the evaluation of aptamer-protein interaction. So far, much fewer algorithms for protein-DNA docking have been specifically developed. The fast Fourier correlation techniques calculate shape complementarity and are mainly used in the protein-protein docking field, which are applied in the computational study of protein/DNA complexes [26]. The ZDOCK used in this study is also a fast Fourier transform based docking algorithm that searches all possible binding modes of complexes and includes the pairwise shape complementarity in the evaluation. Although the results of simulation and experiment are not fully consistent, the experimental results show that most of these selected aptamer sequences can react with PSA and produce significant signal changes in the frequency of QCM chip. In the study reported by Bini's group [11], they found the computational approach partially confirmed results observed in SELEX that the TBA binding score corresponded to only 81.3% of the best candidate. We think that the information provided from molecular simulations is still valuable to combine with experiments for the selection of aptamers.

In order to obtain better simulation results of protein-nucleic acid interactions, some researchers are dedicated to developing new docking algorithms for protein-DNA complexes. Banitt and Wolfson [27] reported a novel protein-DNA docking algorithm, named as ParaDock, for docking short DNA fragments to the protein based on geometric complementarity in 2011. Another web server for protein-nucleic acid docking called NPDock used specific protein-nucleic acid statistical potentials for scoring and selection of modeled complexes [28]. A distance dependent, knowledge-based coarse grained force field is recently developed by Setny et al. [29] for evaluating protein-DNA docking. The force field can improve the quality of predictions in the protein-DNA docking, and they find shape complementarity and sequence-dependent DNA internal energy have great contribution to the specific protein-DNA interaction.

Besides, we supposed there were another two reasons to explain the inconsistency between the simulation and experimental results: (1) The modeling methods used here can build acceptable and reasonable models of single-strand DNAs, but the DNA model may not fully present the actual situation. (2) In the measurements of biosensors, the immobilization of aptamer on sensor surface may influence the interaction between the aptamer and the target protein. For the first point, the RNAComposer web server could generate the model of RNA automatically combined with the information of secondary structure of DNA sequence predicted by RNAfold web server. In order to build the model of DNA aptamer, we had ever considered using other software or web servers, like the 3D-DART web server [30]. However, users needed to input some structural parameters for building the model of single-stranded DNA, such as the nucleic type (A-form or B-form), global and local bend-angle, and location of bend-angle. The technique for fully automated prediction of DNA 3D structures is still absent so far. Although we edited the atoms of models generated from RNAComposer web server to make the RNA model become a DNA model, we supposed that the DNA model compared with the three-dimensional structure of DNA in real situation might be slightly different. Nevertheless, we consider this approach is able to build an acceptable and reasonable model of single-strand DNA based on the currently available tools. The limitation caused by the correctness of DNA model may be a possible reason to explain that the results of simulation studies are not fully consistent with experimental results.

The second point is an unavoidable issue between the experiment and simulation. The orientation of immobilized aptamer on the sensor surface may influence the interaction with the target protein. In our previous study [22], we used the RNAComposer web server to generate models of RNA sequences for studying the RNA-protein interaction. We also found that our experimental findings on the biosensor were not fully consistent with the computational results. On the other hand, the detection scheme on biosensors is also commonly used in the enzyme-linked immunosorbent assay (ELISA) except that no labeled secondary probe agent is used. The measured signals on biosensors can present the potentials of aptamers in the medical applications.

### 3.3. Second-Generation Sequence Calculations and Molecular Simulation Results

On the basis of the QCM experimental results of the first-generation sequences, we selected PSAG15, PSAG112, PSAG117, and PSAG119 as the parent sequences to generate second-generation sequences. Similar to the calculations on the first-generation sequences, the process for producing second-generation sequences was made using the genetic algorithm, which produced 12 sequences (as shown in Table 3). The secondary structure of the sequences was analyzed using the RNAfold web server. According to the analysis results of the sequences, PSAG27 and PSAG211 were removed. For other sequences, DNA molecular models were built using the RNAComposer web server, and the results obtained from docking simulations by using ZDOCK were shown in Table 3. The three sequences with the most favorable ZRANK scores, listed in descending order, were PSAG28, PSAG212, and PSAG24. These three sequences were subsequently selected for the next stage of the experiment. A DNA aptamer sequence, ΔPSap4#5, introduced in a study by Anderson and Mecozzi [10] was used as the baseline sequence for comparisons. Simulations and experiments were also performed for the sequence ΔPSap4#5, and this sequence received a ZDOCK and ZRANK score of 45.1 and −70 in the simulations, respectively.

### 3.4. QCM Experimental Results of the Second-Generation Sequences

**Figure 2:**
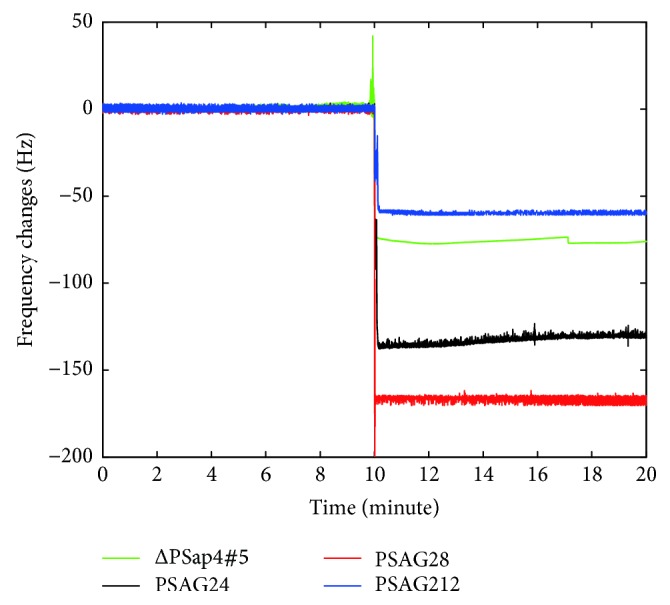
The QCM experiments for the second-generation aptamer sequences. Frequency variations produced by the binding reactions between second-generation sequences and PSA in the QCM experiments.

The second-generation sequences selected were synthesized for a QCM experiment, which produced the results shown in Figure 2. The experiment data are compiled in Table 4. The experiment results showed the amount of signals generated by the second-generation sequences during the experiment, in which only PSAG212 generated a signal smaller than that generated by ΔPSap4#5. Concerning the other two selected sequences, they produced clear signals when interacting with PSA of an identical concentration. In particular, PSAG28 produced an amount of signal which approximately was 2 times higher than that produced by the best sequence ΔPSap4#5 introduced in an existing literature. Besides, PSAG24 could generate an amount of signal that was almost 1.7 times higher than that of ΔPSap4#5.

### 3.5. Measuring the Binding Reactions between Second-Generation Nucleic Acid Aptamer Sequences and PSA by Using the SPR Biosensor

**Figure 3:**
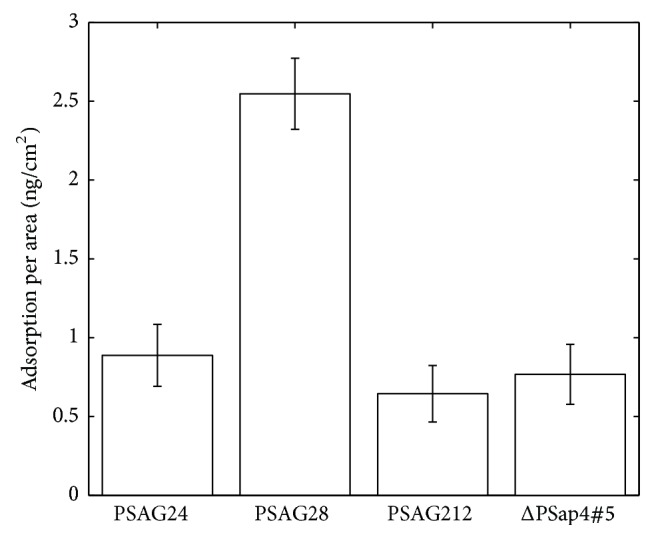
The data of SPR experiments for the second-generation aptamer sequences. Binding reactions between different nucleic acid aptamer sequences and PSA measured by using the surface plasmon resonance biosensor.

To verify the binding reactions between second-generation nucleic acid aptamer sequences and PSA, we performed an experiment by using the SPR biosensor. The SPR experiment results are shown in Figure 3, which reveals that the largest binding amount was produced by the interaction between PSA and PSAG28 immobilized on the sensor surface. The amount of change in SPR signal was equivalent to a biomolecular surface coverage of 2.55 ± 0.23 ng/mm^2^ on the sensor surface. A largest binding amount was also generated when PSA reacted with PSAG24 fixed on the surface; the amount of change in SPR signal was equivalent to a change of 0.89 ± 0.2 ng/cm^2^ in the biomolecular surface coverage. Regarding ΔPSap4#5, it induced a change of 0.77 ± 0.19 ng/cm^2^ in the experiments. PSAG212 displayed the worst performance, generating a change of 0.65 ± 0.18 ng/cm^2^ in the biomolecular surface coverage. In essence, the SPR experiment results were consistent with those obtained using the QCM.

**Figure 4:**
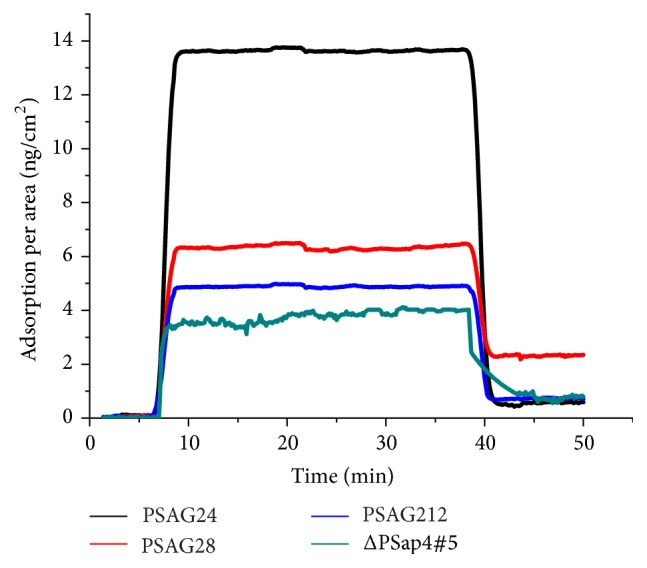
SPR sensorgrams. Representative SPR curves for these four kinds of aptamer-PSA interactions.

The kinetic parameters of these binding reactions were calculated from global fitting of the SPR sensorgram data. Representative SPR curves for these aptamer-PSA interactions are shown in Figure 4. It was worth noting that PSAG24 could produce a large and apparent signal in the association phase of reaction in the sensorgram, but the signal decreased dramatically in the dissociation stage.  Table 5 shows the kinetic parameters for these four kinds of aptamer-PSA interactions. According to the values binding affinity (*K*_*A*_), the sequences had the most favorable binding affinity with PSA, listed in descending order, being PSAG28, PSap4#5, PSAG24, and PSAG212. PSAG24 could produce a slightly larger amount of change in the average SPR signal than that of ΔPSap4#5 (shown in Figure 3), yet the binding ability of ΔPSap4#5 to PSA was better in the viewpoint of kinetic parameters. Concerning the amount of SPR signal created by the binding reactions, that of PSAG28 was almost 3 times higher than that of ΔPSap4#5. PSAG28 even showed superior kinetic parameters compared with other two aptamers selected from the second-generation sequences and ΔPSap4#5. Thus, we successfully selected an aptamer with high affinity for PSA by using the combination of computational approaches and biosensing technologies in this study. We consider that PSAG28 is a suitable aptamer for molecular recognition element in the aptasensors, and it also has potential in other biomedical applications.

## 4. Conclusions

In this study, structural analyses, molecular simulations, and biosensor experiments were used to identify DNA aptamers that featured high binding affinity with PSA. By using nucleic acid aptamer sequence analysis tools such as RNAfold web server, sequences that did not form a clear secondary structure could be eliminated. Furthermore, molecular simulation results gave us information to exclude aptamers that were predicted with poor binding to PSA from aptamer candidates, which effectively reduced the number of experiments in the aptamer selection. Through the screening, we successfully found one sequence (i.e., PSAG28) that could produce a relatively superior binding reaction with PSA. This aptamer showed almost 3-fold higher binding signal in the SPR experiment than that of the best PSA-binding aptamer reported previously, ΔPSap4#5. Besides, kinetic parameters for the evaluation of interaction between the aptamer and PSA also demonstrated that PSAG28 was the best aptamer. This study reveals that the computational approach is valuable for use in the post-SELEX screening procedure for facilitating the preselection of aptamer candidates that will be tested in the further screening experiments. For getting much better simulation results, the tool that can predict and model the 3D structures of single-stranded DNA sequences precisely and automatically is still a strong demand.

## Supplementary Material

The detailed information of generating new mutated sequences by programs and modifying the atoms in the molecular model of nucleic acid are provided in section S1.1 and Figure S1, respectively.

## Figures and Tables

**Table 1:** First-generation sequences and results from the analysis and calculations.

Name	Sequence (5′-3′)	Minimum free energy (kcal/mol)	Dot-bracket format	ZDOCK score	ZRANK score	Rank
PSAG11	**TTTTT**CTGTTGCCCGGAACGTCGTGGCCC**TTT**	−5.6	..........((((((....))).))).....	17.34	−81.44	1
PSAG12	**TTTTT**GTGGTGTTTATTGTTTACTGTCCC**TTT**	−0.2	.......((((.........))))........	49.27	−60.91	
PSAG13	**TTTTT**CTGGTGTTTATTCCATCAAATATC**TTT**	−3.1	.......(((((((........)))))))...	40.03	−60.33	
PSAG14	**TTTTT**AATATCAACTTGGTTTACTGTCCC**TTT**	−0.2	................((........))....	43.93	−70.08	
PSAG15	**TTTTT**CTGGAATGATTTCCCGGTTGTCTC**TTT**	−3.3	....((.((((....)))).))..........	38.78	−72.4	7
PSAG16	**TTTTT**ACTTTGTTTATTGTTTACTGTCCC**TTT**	0	................................	N.A.	N.A.	
PSAG17	**TTTTT**CTGGTCCGGGTACGTTTTTTGGCC**TTT**	−4.8	.......((.(((((.......)))))))...	39.73	−71.49	8
PSAG18	**TTTTT**CCGCAGTTTATTGTTTACTGTCCC**TTT**	−3.5	.......(((((.........)))))......	40.05	−67.55	
PSAG19	**TTTTT**GTGTTGCCCGGAACGTCGTATATC**TTT**	−0.8	....((((((......)))).)).........	48.16	−68.89	
PSAG110	**TTTTT**AATATCAACTTGCCATCAAGGCCC**TTT**	−3.3	................(((.....))).....	45.01	−68.88	
PSAG111	**TTTTT**GTGTTGCCATTTCCCGGTTGTCTC**TTT**	−1.3	..........(((.......))).........	48.6	−64.73	
PSAG112	**TTTTT**ACTTAATGCGGAACGTCGTGGCCC**TTT**	−1.4	............((((....))))........	42.58	−76.89	5
PSAG113	**TTTTT**GTGTTGCCCGGAACTTTTTTGGCC**TTT**	−3	..........(((.(((....))).)))....	39.77	−67.01	
PSAG114	**TTTTT**CCGCACCGGGTACGGTCGTGGCCC**TTT**	−10.8	.....((((((((....)))).))))......	43.45	−78.91	2
PSAG115	**TTTTT**AATATCAACTTGCCAGGTTGTCTC**TTT**	−2.7	..........((((((...)))))).......	44.36	−61.84	
PSAG116	**TTTTT**ACTTAATGATTTCCCTCAAATATC**TTT**	0	................................	N.A.	N.A.	
PSAG117	**TTTTT**AATATCCGGGTACGTTTTTTGGCC**TTT**	−2.4	............((((.((.....))))))..	47.52	−74.57	6
PSAG118	**TTTTT**CCGCACAACTTGCCATCAAATATC**TTT**	−1.1	.......(((.....)))..............	46.13	−76.9	4
PSAG119	**TTTTT**ACGCACCGGGTACGTTTTTTGGCC**TTT**	−2.4	............((((.((.....))))))..	40.41	−77.84	3
PSAG120	**TTTTT**CCTTAATGATTTCCCGGTTGTCTC**TTT**	0	................................	N.A.	N.A.	

**Table 2:** Selected first-generation sequences and their binding reactions with the PSA, as measured by the QCM.

Name	Average and standard deviation values of frequency changes	Ranked results in experiments	Ranked results in simulations
PSAG11	19.2 ± 1.7	8	1
PSAG15	108.2 ± 14	1	7
PSAG17	37.3 ± 1.9	6	8
PSAG112	71.7 ± 1.3	4	5
PSAG114	63 ± 5.4	5	2
PSAG117	82.9 ± 5.8	3	6
PSAG118	24.9 ± 3.9	7	4
PSAG119	91.3 ± 9.7	2	3

**Table 3:** Second-generation sequences and analysis and calculation results.

Name	Sequence (5′-3′)	Minimum free energy (kcal/mol)	Dot-bracket format	ZDOCK score	ZRANK score	Rank
PSAG21	**TTTTT**CTGGAATGATTAACGTCGTGGCCC**TTT**	−3.3	.......((.(((((....)))))..))....	43.44	−68.68	
PSAG22	**TTTTT**ACTTAATGCGGTCCCGGGGGGCTC**TTT**	−4.9	..............(((((....)))))....	45.77	−69.16	
PSAG23	**TTTTT**CTGGAATGATTACGTTTTTTGGCC**TTT**	−1	.......((((((....)))))).........	41.61	−56.12	
PSAG24	**TTTTT**AATATCCGGGTTCCCGGTTGTCTC**TTT**	−6.7	......(((.((((....)))).)))......	17.54	−70.91	3
PSAG25	**TTTTT**CTGGAATGATTTCCCGGTTTGGCC**TTT**	−4.3	.......((((....)))).(((...)))...	45.69	−55.04	
PSAG26	**TTTTT**ACGCACCGGGTACGTTTTTGTCTC**TTT**	−2.6	............(((.(((....))))))...	44.63	−60.12	
PSAG27	**TTTTT**ACTTAATGCGTACGTTTTTTGGCC**TTT**	0	................................	N.A.	N.A.	
PSAG28	**TTTTT**AATATCCGGGGAACGTCGTGGCCC**TTT**	−4.1	............(((..((...))..)))…	42.64	−86	1
PSAG29	**TTTTT**ACTTAATGCGGACGTTTTTTGGCC**TTT**	−1.7	............((.((......)).))....	44.51	−65.83	
PSAG210	**TTTTT**ACGCACCGGGTAACGTCGTGGCCC**TTT**	−5.4	............((((.((...)).))))...	42.61	−68.5	
PSAG211	**TTTTT**ACTTAATGAATACGTTTTTTGGCC**TTT**	0	................................	N.A.	N.A.	
PSAG212	**TTTTT**ACGCACCGGGTACGTTTTTTGGCC**TTT**	−2.4	............((((.((.....))))))..	40.41	−77.84	2

**Table 4:** Selected second-generation sequences and their binding reactions with the PSA in QCM measurements.

Name of aptamer	Average and standard deviation values of frequency changes (Hz)	Ranked results in experiments
PSAG24	131.3 ± 4.9	2
PSAG28	166.2 ± 6.7	1
PSAG212	59.5 ± 5.8	4
ΔPSap4#5^*∗*^	76.1 ± 2.1	3

^*∗*^A sequence introduced in a study by Savory et al. [14] that showed high affinity with PSA.

**Table 5:** Kinetic parameters of aptamer-PSA interactions by fitting SPR sensorgrams.

Name of aptamer	*k*_*a*_ (×10^3^ M^−1^s^−1^)	*k*_*d*_ (×10^−3^ s^−1^)	*K*_*A*_ (×10^6^ M^−1^)
PSAG24	3.37 ± 0.62	12.34 ± 0.58	0.27 ± 0.04
PSAG28	6.62 ± 0.77	6.7 ± 0.37	0.99 ± 0.06
PSAG212	3.11 ± 0.5	11.23 ± 0.8	0.28 ± 0.03
ΔPSap4#5	4.2 ± 0.62	7.08 ± 0.23	0.59 ± 0.07
