# Supplementary material for: The Combination of Computational and Biosensing Technologies for Selecting Aptamer against Prostate Specific Antigen
Source: Biomed Res Int. 2017 Mar 28;2017:5041683. doi: 10.1155/2017/5041683 (PMC5387809; doi:10.1155/2017/5041683)
Supplement: Supplementary file 1 — The detailed information of generating new mutated sequences by programs and modifying the atoms in the molecular model of nucleic acid are provided in section S1.1 and Figure S1, respectively. [file 5041683.f1.pdf]

Supplementary Materials

The combination of computational and biosensing technologies for selecting aptamer against prostate specific antigen

Pi-Chou Hsieh [1], Hui-Ting Lin [2], Wen-Yih Chen [3], Jeffrey J. P. Tsai [1], Wen-Pin Hu [1,4,*]

[1] Department of Bioinformatics and Medical Engineering, Asia University, Taichung City 41354, Taiwan

[2] Department of Physical Therapy, I-Shou University, Kaohsiung City 82445, Taiwan

[3] Department of Chemical and Materials Engineering, National Central University, Jhong-Li 32001, Taiwan

[4] Department of Medical Laboratory Science and Biotechnology, China Medical University, Taichung City 40402, Taiwan

The appropriate subject category:

Immunologicl Procedures, DNA Recombinant Techniques and Nucleic Acids

* Corresponding author: Wen-Pin Hu

TEL:+886-4-23323456 ext.20022

FAX: +886-4-23320718

Email address: wenpinhu@asia.edu.tw (W.-P. Hu)

S1. Materials and Methods

S1.1 Computational process by using the genetic algorithm.

The Genetic Algorithm Toolbox for MATLAB® was downloaded from this link: http://codem.group.shef.ac.uk/index.php/ga-toolbox. The toolbox was developed at the Department of Automatic Control and Systems Engineering of The University of Sheffield, UK and the software version was v1.2. After unzipping the download file, users have to correct the filename extension of these files from M to m. After this modification, the function named as xovsp, which is a low-level crossover function, can be used to generate new sequences based on the input sequence data. The XOVR parameter in this function was set by using the default value. Bases A, T, G, and C for sequences were numbered 00, 01, 10, and 11, respectively. Here, we use four short-length DNA sequences to illustrate the processes.

1. Original sequences:
   CTGGTGTTTA, GTGTTGCCCG, AATATCAACT, ACTTAATGAT

2. Convert sequences according to the codes
   11011010011001010100, 10011001011011111110, 00000100011100001101, 00110101000001100001

3. Run the Matlab function: xovsp
   Four new sequences will be produced for each run.

```
OldChrom = [ 1 1 0 1 1 0 1 0 0 1 1 0 0 1 0 1 0 1 0 0;
             1 0 0 1 1 0 0 1 0 1 1 0 1 1 1 1 1 1 1 0;
             0 0 0 0 0 1 0 0 0 1 1 1 0 0 0 0 1 1 0 1;
             0 0 1 1 0 1 0 1 0 0 0 0 0 1 1 0 0 0 0 1];
NewChrom = xvosp(OldChrom);
```

4. The data of NewChrom:

```
1 1 0 1 1 0 0 1 0 1 1 0 1 1 1 1 1 1 1 0
1 0 0 1 1 0 1 0 0 1 1 0 0 1 0 1 0 1 0 0
0 0 0 0 0 1 0 1 0 0 0 0 0 1 1 0 0 0 0 1
0 0 1 1 0 1 0 0 0 1 1 1 0 0 0 0 1 1 0 1
```

5. By using a self-written C# program for the conversion of data to sequence information, four new sequences are obtained.
   CTGTTGCCCG, GTGGTGTTTA, AATTAATGAT, ACTATCAACT

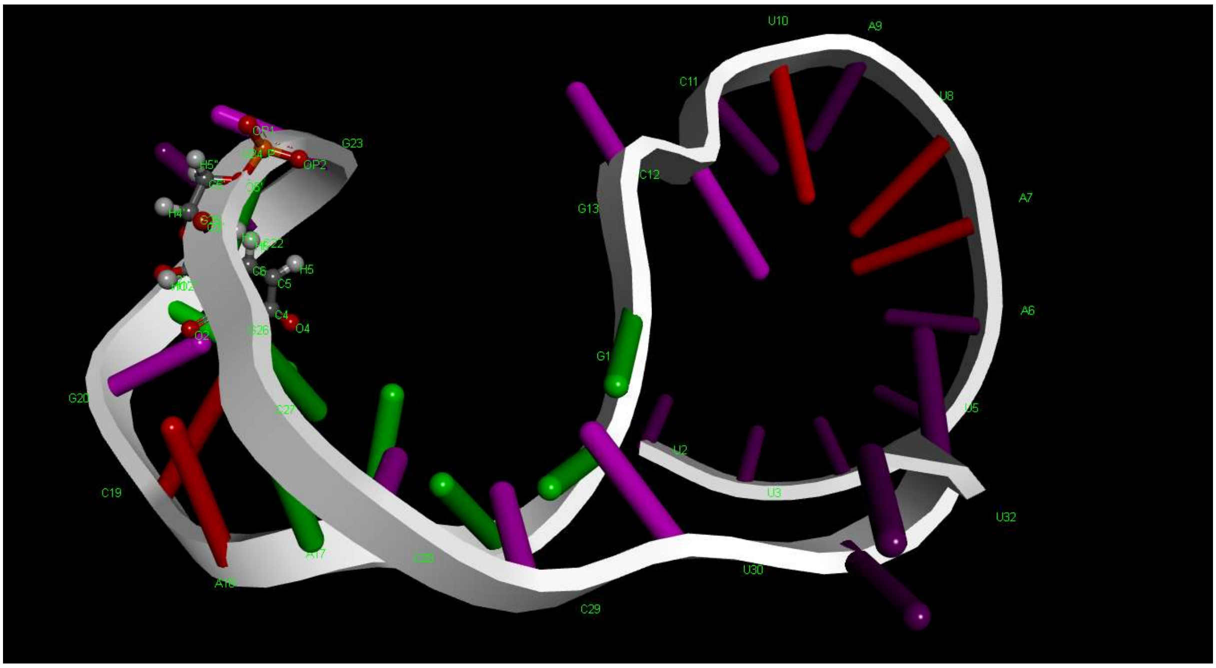

(A)

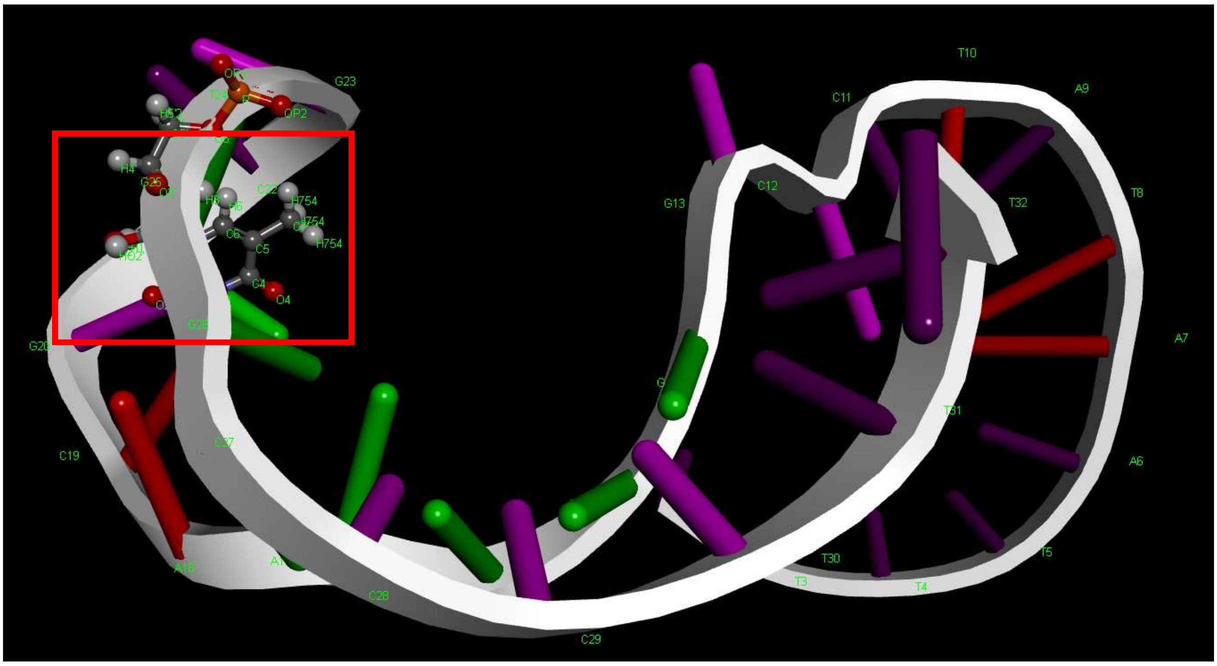

(B)

Figure S1. Model of the 3D Structure of PSAG28. (A)This RNA model is generated by using the RNAComposer web server and viewed in the Accelrys Discovery Studio (DS). (B) The structure of uracil was edited by DS software to become the structure of thymidine. Please note the structure inside the inserted red rectangle. The hydrogen atom at the C5 position was changed to CH_3.
